# Aging and caloric restriction impact adipose tissue, adiponectin, and circulating lipids

**DOI:** 10.1111/acel.12575

**Published:** 2017-02-03

**Authors:** Karl N. Miller, Maggie S. Burhans, Josef P. Clark, Porsha R. Howell, Michael A. Polewski, Tyler M. DeMuth, Kevin W. Eliceiri, Mary J. Lindstrom, James M. Ntambi, Rozalyn M. Anderson

**Affiliations:** ^1^ Division of GeriatricsDepartment of MedicineSMPHMadisonWI53706USA; ^2^ Department of Nutritional SciencesUniversity of Wisconsin MadisonMadisonWI53706USA; ^3^ Laboratory for Optical and Computational InstrumentationUniversity of Wisconsin MadisonMadisonWI53706USA; ^4^ Department of Biostatistics and Medical InformaticsUniversity of WisconsinMadisonWI53705USA; ^5^ Department of BiochemistryUniversity of WisconsinMadisonWI53706USA; ^6^ GRECCWilliam S. Middleton Memorial Veterans HospitalMadisonWI53705USA; ^7^ Present address: Fred Hutchinson Cancer CenterSeattleWAUSA

**Keywords:** adiponectin, adipose tissue, aging, caloric restriction, fatty acids, NAD metabolism

## Abstract

Adipose tissue expansion has been associated with system‐wide metabolic dysfunction and increased vulnerability to diabetes, cancer, and cardiovascular disease. A reduction in adiposity is a hallmark of caloric restriction (CR), an intervention that extends longevity and delays the onset of these same age‐related conditions. Despite these parallels, the role of adipose tissue in coordinating the metabolism of aging is poorly defined. Here, we show that adipose tissue metabolism and secretory profiles change with age and are responsive to CR. We conducted a cross‐sectional study of CR in adult, late‐middle‐aged, and advanced‐aged mice. Adiposity and the relationship between adiposity and circulating levels of the adipose‐derived peptide hormone adiponectin were age‐sensitive. CR impacted adiposity but only levels of the high molecular weight isoform of adiponectin responded to CR. Activators of metabolism including PGC‐1a, SIRT1, and NAMPT were differentially expressed with CR in adipose tissues. Although age had a significant impact on NAD metabolism, as detected by biochemical assay and multiphoton imaging, the impact of CR was subtle and related to differences in reliance on oxidative metabolism. The impact of age on circulating lipids was limited to composition of circulating phospholipids. In contrast, the impact of CR was detected in all lipid classes regardless of age, suggesting a profound difference in lipid metabolism. These data demonstrate that aspects of adipose tissue metabolism are life phase specific and that CR is associated with a distinct metabolic state, suggesting that adipose tissue signaling presents a suitable target for interventions to delay aging.

## Introduction

It has been long established that aging is the greatest risk factor for a range of diseases including diabetes, cancer, cardiovascular disease, and neurodegenerative disease (Lopez‐Otin *et al*., 2013; Kennedy *et al*., 2014). Caloric restriction (CR) is a dietary intervention that delays aging and extends the period of health in diverse species (Anderson & Weindruch, 2010). One of the hallmarks of caloric restriction is the marked reduction in adiposity, a consequence that may be important in the mechanisms of CR given the endocrine function of adipose tissue. Adipokines and lipokines secreted from white adipose tissue impact peripheral tissue fuel utilization and the balance of energy generation from lipid or carbohydrate sources (Lago *et al*., 2007; Sethi & Vidal‐Puig, 2007; Ouchi *et al*., 2011). However, it is unclear what effect aging has on adipose tissue metabolic integrity and how that relates to secretion of systemic regulatory factors. Prior studies of gene expression in adipose tissues from old rats and adult mice show that CR induces expression of genes involved in multiple aspects of metabolism. A further difference includes the increased circulating levels of the adipose tissue‐derived peptide hormone adiponectin with long‐term stringent (40%) CR (Combs *et al*., 2003; Zhu *et al*., 2004). Adiponectin circulates as a multimer, activates lipid metabolism in target tissues, and is associated with increased insulin sensitivity (Turer & Scherer, 2012). Assembly into a high molecular weight form (HMW) is essential to adiponectin function (Waki *et al*., 2003), and although total adiponectin levels are not changed with aging (Combs *et al*., 2003), the impact of age and modest CR on HMW adiponectin has not been established. In addition to peptide factors, adipose tissues also secrete free fatty acids, some of which may act as signaling molecules in metabolic homeostasis (Cao *et al*., 2008). The impact of age and modest CR on the adipose tissue‐derived serum lipid profile has not been reported.

In order to understand whether age‐related changes in adiposity are associated with a change in adipose tissue function, we undertook a cross‐sectional mouse study focusing on adipose tissue metabolism and circulating levels of adipose tissue‐derived signaling molecules. To capture the trajectory of aging, the study involved adult, late‐middle‐aged, and advanced‐aged C3B6F1 hybrid mice. Parallel groups of mice on modest (16%) CR taken at each age served to uncover aspects of adipose tissue aging that were responsive to delayed aging. We investigated the relationship between adiposity, adipocyte size, and adiponectin levels at three age groups of mice on control or CR diets. We determined whether differences with age and diet were associated with changes in factors downstream of adiponectin and factors that connect with adiponectin signaling including NAD metabolism. To investigate differences in adipose tissue lipid metabolism, we profiled serum lipids including free fatty acids that are derived from adipose tissue. The goal of these studies was to determine how age and CR impacted adipose tissue function beyond simple differences in adiposity and whether relationships between adipocyte size and secretory profiles were sustained with age or altered with CR.

## Results

### Age and CR impact adiposity and adiponectin production

**Figure 1:**
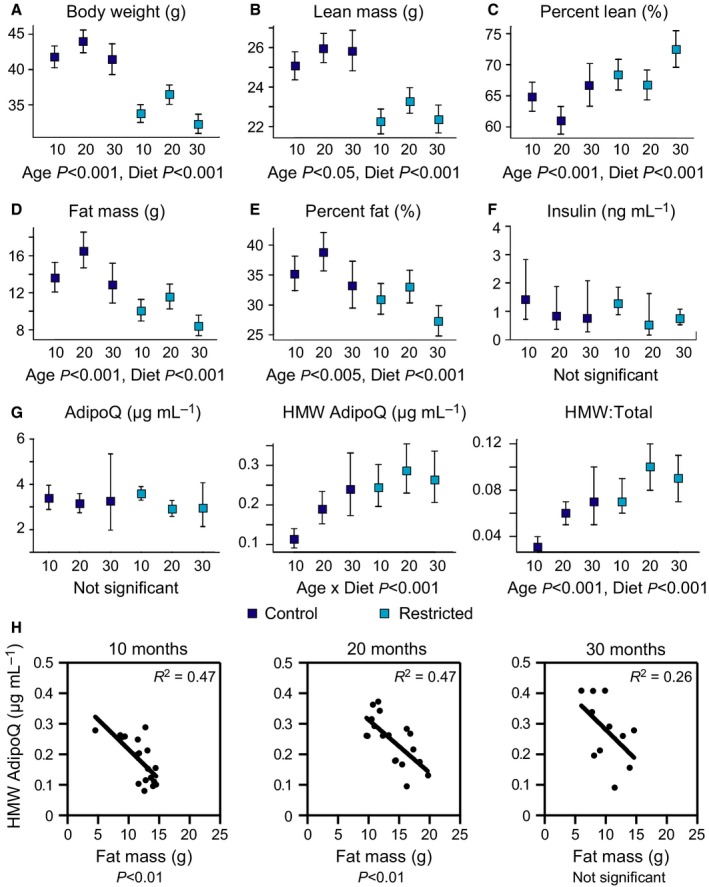
Impact of age and diet on mouse body composition, fasting insulin, and adiponectin. (A–D) Body weight and DEXA estimates of lean mass, percent lean mass, fat mass, and percent fat mass as a function of age and diet (*n* = 5–10 per group). (E) ELISA detection of serum insulin levels (*n* = 3–6 per group). (F) ELISA detection of serum adiponectin levels (*n* = 4–9 per group). (G) Linear regression of HMW adiponectin and fat mass at each age (*n* = 11–18 per group). Data are shown as means ± 95% CI. HMW: high molecular weight. Significance determined by two‐way ANOVA.

Cohorts of male mice (C3B6F1 hybrid) were established for a cross‐sectional study that included animals at 10, 20, and 30 months of age (*n* = 10–11 per group, per diet), representing adult, late‐middle age, and advanced age for this strain of mice. Younger animals were not included to avoid the contributions of growth and development to differences among age groups. For the aging study, mice were fed a control diet of 87 kcal week^−1^. This level of calorie intake is ~95% of *ad libitum* for this strain, where all the mice eat all the food so that precise food intake is known. This strategy of controlled feeding has the further advantage of avoiding obesity. A second cohort of mice were placed on a CR diet of 73 kcal week^−1^ (16% restriction from control) from 2 months of age and harvested at the same time points indicated above. Survival of the controls was ~45% at 30 months (5/11 mice remaining), consistent with the expected lifespan for this strain, and ~73% for the mice on CR (8/11 mice remaining). Two‐way ANOVA reveals significant effects of age and diet for body composition (Fig. 1A). Body weight, percent lean, and percent fat mass estimates measured by dual‐energy X‐ray absorptiometry were highest in 20‐month‐old mice, with significant main effects of age and diet detected in all parameters (Fig. 1B–E). As expected, body weight, lean mass, and fat mass were all lower in CR animals compared to controls; however, percent lean mass was significantly higher and adiposity by both metrics significantly lower with CR at each time point.

Loss of insulin sensitivity is widely considered to be a contributing factor in the development of pathologies associated with aging (Facchini *et al*., 2001; Russell & Kahn, 2007). An impact of age on fasting serum insulin was not detected in this study in control animals (Fig. 1F), aligning with similar reports in rats and mice (Barnard *et al*., 1995; McCarter *et al*., 2007). Furthermore, fasting serum insulin was not significantly different between CR and control animals at any time point, perhaps due to the modest level of CR implemented in this study and the fact that the controls were not *ad libitum* fed. Neither age nor diet had a significant effect on total circulating adiponectin levels (Fig. 1G); however, an interaction between age and diet was detected for levels of the high molecular weight (HMW) isoform, and both age and diet effects were detected for the ratio of HMW to total adiponectin that was significantly higher in CR animals.

Previous reports have documented the negative correlation between adiposity and circulating levels of adiponectin (Arita *et al*., 1999; Turer *et al*., 2011). To understand whether this relationship holds in the absence of obesity and whether there is an impact of age, regression analysis was conducted using data from all control and CR mice of 10, 20, and 30 months of age. Levels of total adiponectin were not correlated with adiposity as measured by mass in grams or percent body weight for any age group in the study (Fig. S1, Supporting information). A significant inverse relationship between HMW adiponectin and fat mass was identified in mice of 10 or 20 months of age, but not at 30 months of age (Fig. 1H). These data indicate that in lean animals, the levels of HMW, but not total adiponectin, are related to adiposity and that this relationship is sensitive to age.

### Age and CR impact the association between adipocyte size and adiponectin production

**Figure 2:**
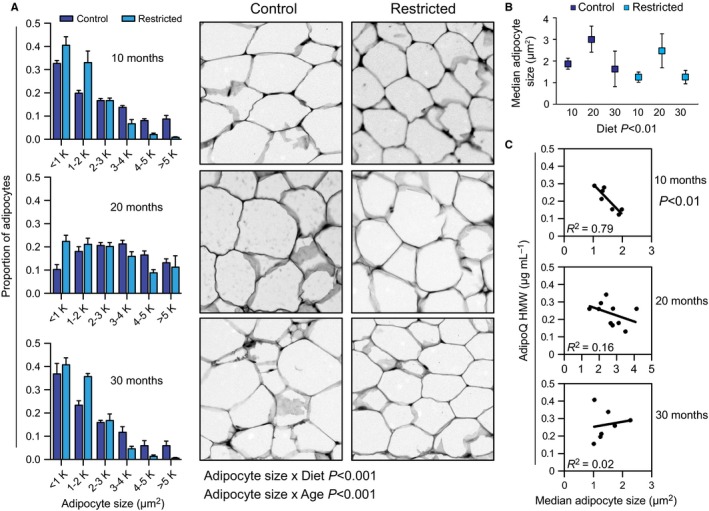
Changes with adipose tissue morphology with age and CR. (A) Distributions of adipocyte size and representative images of hematoxylin and eosin‐stained adipose sections (grayscaled) (*n* = 4–7 per group). (B) Median adipocyte size (*n* = 4–7 per group). (C) Linear regression of HMW adiponectin and median adipocyte size, combining control and restricted diets (*n* = 7–10 per group). Data are shown as means ± SEM, or means ± 95% CI. HMW: high molecular weight. Significance determined using a mixed‐effects model (A) or two‐way ANOVA (B).

Morphometric analysis was conducted on fixed paraffin‐embedded sections from epididymal white adipose tissue. Analysis of log‐transformed data revealed significant main effects of both age and diet on adipocyte size (Fig. 2A). The adipocyte median size and size distribution were largest in the 20‐month‐old animals and were significantly smaller in CR mice compared to controls, regardless of age (Fig. 2B). Circulating levels of total adiponectin were not correlated with either body weight, fat mass, or median adipocyte size (Fig. S2, Supporting information). In contrast, circulating levels of HMW adiponectin correlated with body weight and fat mass, but not median adipocyte size (Fig. S2, Supporting information). Regression analysis conducted on data separated by age category (Fig. 2C) revealed a robust and significant inverse correlation between circulating levels of HMW and median adipocyte size in 10‐month‐old mice, but not in 20‐ or 30‐month‐old mice. These data indicate that the relationship between adiponectin and adiposity, including adipocyte size, is age labile and that in aged animals, the mechanisms linking adiponectin production and processing to adiposity are likely compromised.

### CR impacts levels and modification status of metabolic regulators

**Figure 3:**
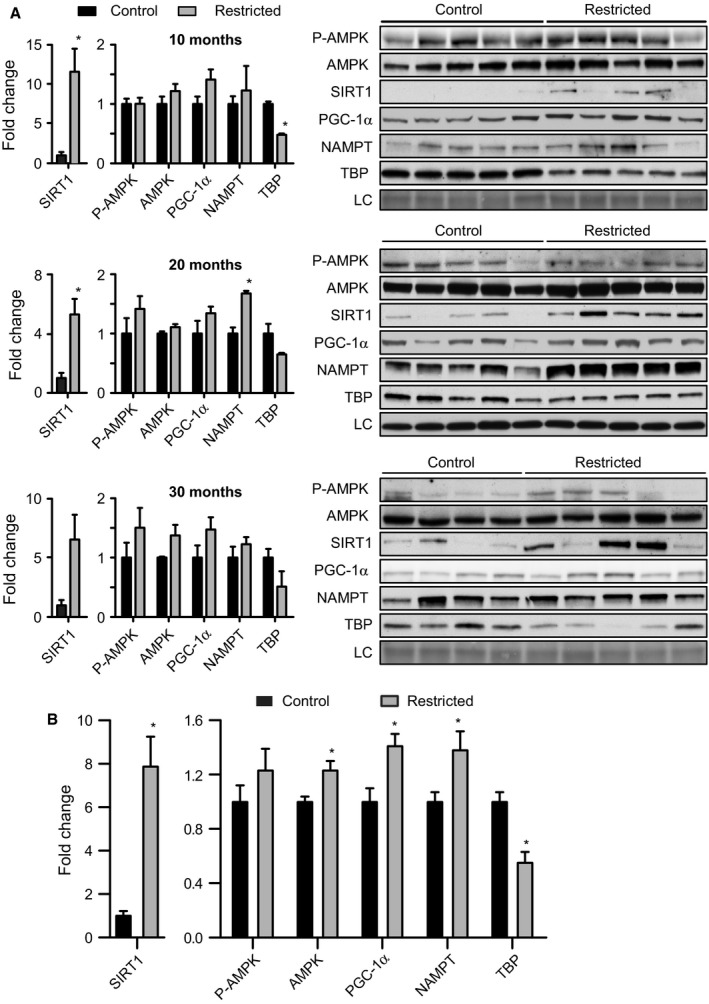
CR activates metabolic regulators in adipose tissue. (A) Detection of levels of indicated proteins (*n* = 4–5 per group). (B) Quantitation of protein levels across all age groups (*n* = 13–15 per group). Data are shown as means ± SEM; asterisk (*) indicates *P* < 0.05. Significance determined by Student's *t*‐test.

We investigated the impact of CR on metabolic regulators downstream of the adiponectin receptor (Iwabu *et al*., 2010) in adipose tissue. These include AMPK, the primary effector of adiponectin signaling (Yamauchi *et al*., 2002), PGC‐1a (peroxisome proliferator‐activated receptor gamma coactivator 1 alpha), a master regulator of both nuclear and mitochondrial encoded genes involved in oxidative phosphorylation (Huss *et al*., 2002; Mootha *et al*., 2003), SIRT1, an NAD‐dependent deacetylase shown to activate PGC‐1a (Nemoto *et al*., 2005; Rodgers *et al*., 2005; Gerhart‐Hines *et al*., 2007), and NAMPT (nicotinamide phosphoribosyltransferase), an enzyme of the NAD salvage pathway that activates SIRT1 and is activated by AMPK (Fulco *et al*., 2008). A consistent increase in PGC‐1a levels was detected in CR tissues compared to controls, but was not significant for any individual age group (Fig. 3A). Analysis of mean adjusted values across age groups revealed a significant ~1.4‐fold increase in PGC‐1a in the CR tissues (Fig. 3B). SIRT1 protein levels were significantly higher in CR adipose tissues at 10 and 20 months of age (Fig. 3A), and a significant increase in levels of SIRT1 was identified across age groups (Fig. 3B). Protein levels of NAMPT in adipose tissues were significantly higher in CR mice compared to controls at 20 months of age, and there was an overall significant increase in NAMPT with CR across age groups. A significant impact of CR to increase levels of AMPK was detected across age groups although the differences were not significant for any one age group. Levels of activating phosphorylation [Thr172] of AMPK although numerically higher in CR tissues compared to controls were not significantly different for any age group. These data are consistent with a model where CR activates PGC‐1a in adipose tissues, potentially through regulatory factors downstream of adiponectin.

The lower adiposity and smaller adipocyte size of CR mice pointed to potential differences in growth signaling. TBP (TATA binding protein) is a general transcription factor required by all three RNA polymerases, and it is required for the response to growth stimuli (Johnson *et al*., 2003). Levels of TBP were significantly lower in adipose tissues from CR age groups, suggesting some degree of attenuated growth. The nutrient‐sensitive kinase mTOR (mechanistic target of rapamycin) is a major growth coordinating kinase and has been implicated in the mechanisms of CR in nonmammalian models (Kapahi *et al*., 2010). mTOR levels were not significantly different in CR adipose tissues at any age, but overall levels of activating phosphorylation [Ser2448] were significantly lower with CR (Fig. S3, Supporting information). GSK3b (glycogen synthase kinase 3 beta) is a key regulator downstream of growth‐promoting pathways including insulin signaling, mTOR signaling, and WNT signaling (Beurel *et al*., 2015). Overall GSK3b levels were not significantly different in CR adipose tissues, but inhibitory phosphorylation [Ser9] was significantly lower. The impact of age and diet on mTOR (lower predicted activity) and GSK3b (higher predicated activity) was not equivalent (Fig. S3, Supporting information), suggesting that the growth signaling response to CR may be pathway specific.

### Cellular redox environment is differentially impacted by age and CR

**Figure 4:**
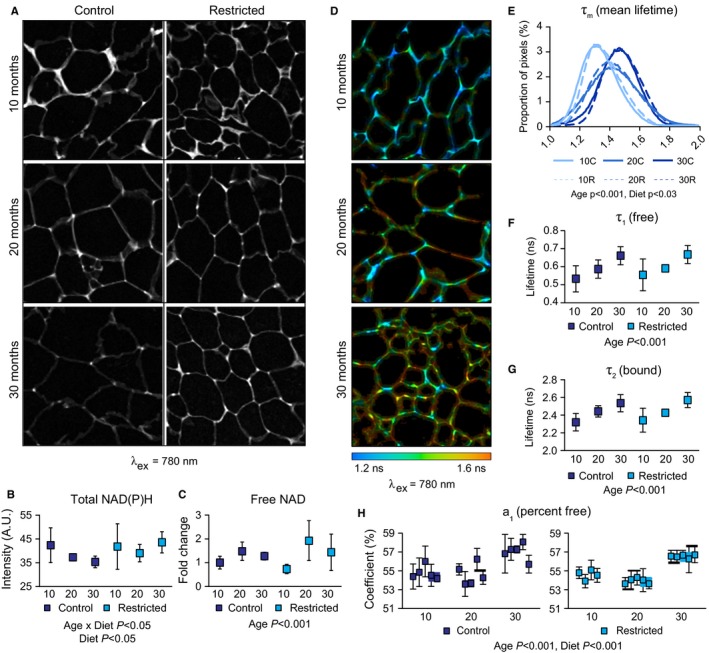
Aging and CR impact NAD(P)H metabolism in adipose tissue. (A) Representative images of NAD(P)H autofluorescence (wavelength of excitation λ_ex_ = 780 nm) and (B) quantitation (*n* = 4–5 per group). (C) Quantitation of free NAD by biochemical assay (*n* = 4–6 per group). (D) Representative images of NAD(P)H mean fluorescence lifetime (τ_m_) (λ_ex_ = 780 nm), (E) τ_m_ distributions, (F) quantitation of τ_1_, (G) τ_2_, (H) a_1_, *n* = 4–5 per group. Data are shown as means ± 95% CI. Significance determined by two‐way ANOVA (B and C) or using a mixed‐effects model (E–H).

To investigate the impact of age and CR on NAD metabolism, we employed a quantitative fluorescence imaging‐based approach that takes advantage of the innate autofluorescence of the nicotinamide ring. This high‐resolution microscopy technique detects levels and chemical properties of the reduced forms of NAD and NADP that can be quantified directly and nondestructively. Multiphoton laser scanning microscopy (MPLSM) quantifies NAD(P)H autofluorescence intensity, informing about total free and bound levels of the cofactors (Denk *et al*., 1990), and fluorescence lifetime imaging microscopy (FLIM) quantifies the kinetics of photon release from the fluorophores, informing about the metabolic environment (Lakowicz *et al*., 1992). To ensure signals captured were derived primarily from adipocytes, heavily vascularized regions of the tissue sections were avoided in image capture and quantification. MPLSM detects NAD(P)H throughout adipocyte cytosolic regions with small areas of intense brightness identified at junctions of three or more adipocytes. An apparent decline in NAD(P)H autofluorescence intensity in control animals did not result in a main effect of age (*P* = 0.08), but a significant impact of CR and an age by diet interaction were detected (Fig. 4A,B). Autofluorescence intensity was significantly higher in adipose tissue from 30‐month‐old CR mice than from controls (*P* < 0.05). Next we used biochemical approach to detect free NAD^+^ and NADH in adipose tissue. In general, levels of total NAD were low in adipose tissues (250 pmol mg^−1^ tissue), and levels of NADH were below the threshold of detection. A significant effect of age was detected where levels of total free NAD were higher in adipose tissues from 20‐month‐old mice than in 10‐month‐old mice and intermediate at 30 months of age (Fig. 4C). An impact of diet on levels of free NAD in adipose tissue was not detected, although this may be due to the modest levels of CR employed in this study.

In order to determine the impact of age and CR on the adipose tissue microenvironment, we measured fluorescence lifetime using FLIM. Mean fluorescence lifetime (τ_m_) is the duration that the NAD(P)H fluorophores stay in the excited state. The kinetics of photon release are characterized by a first‐order decay curve involving a fast component (τ_1_) and a slow component (τ_2_) that correspond to free and protein‐bound pools of NAD(P)H, respectively. Decay values (τ_1_ and τ_2_) are influenced by the immediate local environment including hypoxia, pH, redox, and, in the case of τ_2_, the proteome to which the fluorophores are bound. Decay curves were generated over multiple pulses, repeated for each pixel in the image capture field, and were quantified on a by‐pixel basis and color‐coded according to picoseconds of decay (Figs 4D and S4, Supporting information). Similar to the intensity data, clear differences in τ_m_ were detected in cytosolic regions where three or more adipocytes contact revealing previously unreported metabolic heterogeneity in adipose tissue. Main effects of age and of diet were significant where age shifted τ_m_ to progressively longer values and CR induced a shift longer at each age (Fig. 4E). With age, there was a significant increase in τ_1_ (free) and τ_2_ (bound) that were equivalent in both control and CR tissues (Fig. 4F, G). A main effect of age was detected for the a_1_ coefficient, an indicator of the proportion of free NAD(P)H in the total pool, shifting to greater values. This is consistent with a shift away from oxidative metabolism (Bird *et al*., 2005). A main effect of diet was detected for a_1_ where values were lower with CR, indicative of a more oxidative metabolic state (Fig. 4H). Together these data suggest that aging alters the microenvironment in adipose tissue, changing the intrinsic chemical properties of NAD(P)H (τ_1_, τ_2_). CR does not impact these age‐related changes in the microenvironment, but shifts metabolism toward a more oxidative profile.

### Age and CR impact the fatty acid composition of circulating lipids

**Figure 5:**
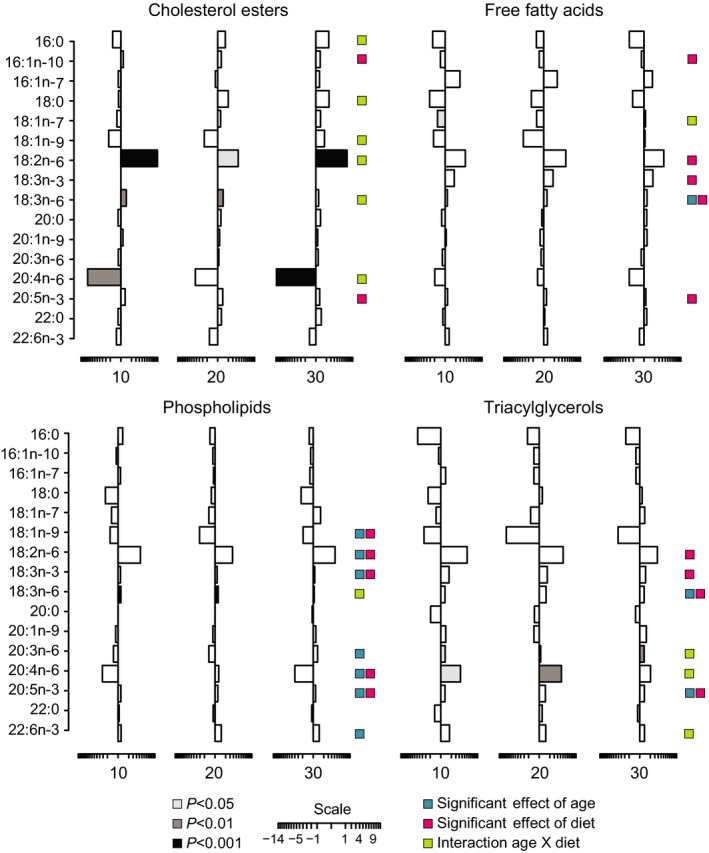
Impact of age and CR on serum fatty acid composition. Serum fatty acid profiles represented as difference in percent abundance (restricted – control) *n* = 5 per diet per age group. Statistical significance determined by Student's *t*‐test (grayscale) or two‐way ANOVA (color‐coded).

Apart from free fatty acids that are adipose tissue derived, serum lipids are generally found complexed as lipoproteins that are liver derived but peripheral tissue depleted. To investigate the fatty acid composition of circulating lipids, we determined chain length and degree of saturation of fatty acids from triglycerides (TG), cholesterol esters (CE), phospholipids (PL), and nonesterified free fatty acids (FFA) by gas chromatography (Tables S1–S4, Supporting information). Differences in relative fatty acid levels between control and CR were calculated for each lipid class at all ages (Fig. 5). The impact of CR on fatty acid species was lipid class specific, but was consistent across age groups and within lipid class. For CE, several monounsaturated fatty acids (MUFA), polyunsaturated fatty acids (PUFA), and saturated fatty acids (SFA) were significantly different with CR (shaded bars, Fig. 5), in contrast to the limited number of differences within FFA, PL, and TG classes, although PUFA were the predominant fatty acid species responding to CR (Fig. 5). Two‐way ANOVA was performed to determine main effects of diet or age or diet by age interactions. PL featured prominently among those species showing a main effect of age (blue boxes, Fig. 5). With the exception of one MUFA, age exclusively affected relative levels of PUFAs. There was a significant main effect of diet on fatty acid composition in all lipid classes analyzed (pink boxes, Fig. 5). Significant age by diet interactions were identified for several fatty acid species in CE with fewer in TG, PL, and FFA classes (green boxes, Fig. 5). Although fatty acid composition was identical for the control and CR diets (Table S5, Supporting information), difference in ratios of circulating essential fatty acids was not equivalent among classes (Fig. S5, Supporting information), suggesting that CR is associated with a change in the underlying lipid metabolism. This concept is supported by the identification of main effects of diet on elongation and saturation indices for FFA, PL, and TG lipid classes (Fig. S6, Supporting information). These data suggest that diet has a greater impact than age on circulating fatty acid composition, that SFA are largely refractory to age and diet, and that PUFA are responsive to both age and diet.

### Adipose tissue‐derived fatty acids are responsive to CR

**Figure 6:**
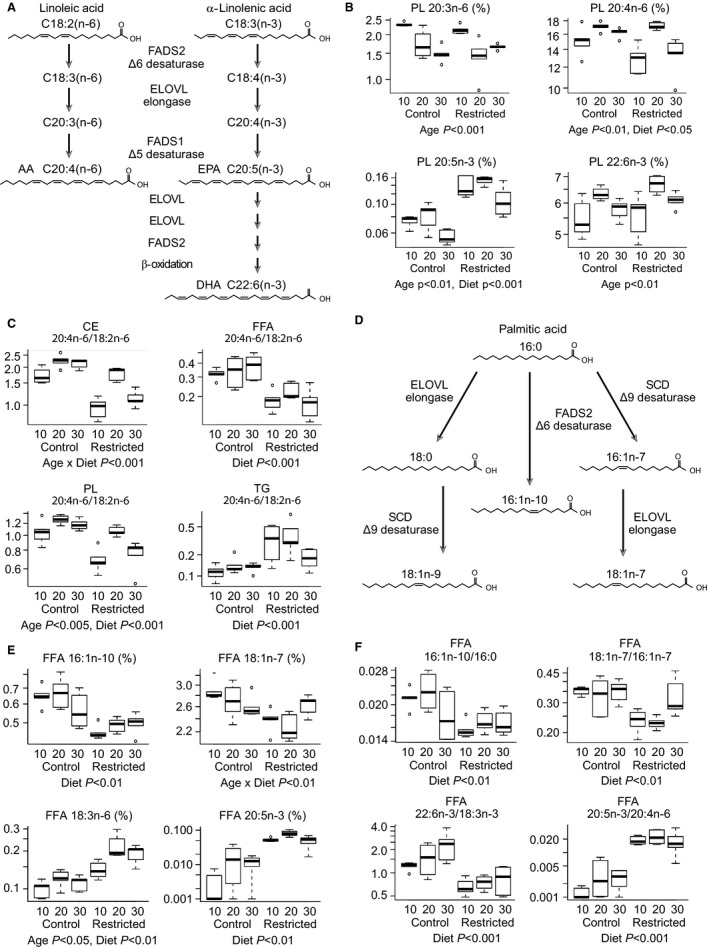
Impact of age and CR on serum poly‐ and mono‐unsaturated fatty acid composition. (A) Schematic of fatty acid synthetic pathways from essential fatty acids. (B) Serum phospholipid fatty acid composition and (C) inflammatory index. (D) Schematic of fatty acid synthesis from palmitate. (E) Effect of age and diet on free fatty acid composition, and (F) elongation, desaturation, and omega 3:6 ratios. Data are shown as medians with IQR (*n* = 5 per group). Statistical significance determined by two‐way ANOVA. CE: cholesterol ester; FFA: free fatty acids; PL: phospholipids; TG: triglycerides. Significance determined by two‐way ANOVA.

Serum lipid composition provides a window into differences in lipid metabolism. Apart from linoleic acid (LA; 18:2n‐6) and alpha‐linolenic acid (18:3n‐3) that are essential fatty acids, all other species are derived from dietary lipids by elongation and desaturation reactions (Fig. 6A). Focusing on age‐sensitive PUFA, in the PL some species showed a biphasic pattern with age. For example, linolenic acid derivative docosahexaenoic acid (DHA; 22:6n‐3) levels were highest at late‐middle age and this pattern was mirrored in eicosapentaenoic acid (EPA; 20:5n‐3), the fatty acid from which it is derived (Fig. 6B). Other species showed linear effects of age. Arachidonic acid (AA; 20:4n‐6) levels increase with age in PL, while levels of the species from which it is derived (20:3n‐6) declined. The impact of CR was species specific; a main effect of diet was detected for AA and EPA with levels higher and lower in serum from CR mice, respectively. For all but the TG, CR induced favorable changes in the ratios of AA to LA, suggesting that there may be underlying differences in the synthesis of lipid‐derived inflammatory mediators in mice on CR (Fig. 6C).

We have shown a significant impact of CR on adipose tissue morphology, adipokine production, and levels of metabolic regulators. These data suggested that CR might also induce differences in adipose‐derived serum lipid signatures. A main effect of diet was detected in FFA MUFA (Fig. 6D) and PUFA (Fig. 6A) species, indicative of pervasive changes in lipid metabolism in adipose tissue (Fig. 6E). Consistent with this, main effects of diet were detected for indices of desaturation (16:1n‐10/16:0), elongation (18:1n‐7/16:1n‐7), and omega 3 (22:6n‐3/18:3n‐3) metabolism that were all lower with CR. The ratio of omega 3 to omega 6 FFA (20:5n‐3/20:4n‐6), which has been used as a positive index of metabolic health, was higher in serum from CR mice (Fig. 6F). This ratio was also higher in serum CE and PL (Fig. S6, Supporting information), but not TG, reminiscent of the inflammatory index shown above. Together, these data suggest that CR impinges on adipose tissue lipid handling resulting in a distinct systemic lipid profile.

## Discussion

Systemic factors that have previously been implicated in aging and CR include insulin and adiponectin. While is clear that lower circulating insulin is associated with enhanced longevity, and that elevated circulating insulin is associated with reduced longevity, the role of insulin in aging per se is less well established. Prior reports of data from rats and mice have indicated that insulin is not different with age (Barnard *et al*., 1995; McCarter *et al*., 2007), consistent with data reported here. Unexpectedly, CR at the level employed in this study had no impact on fasted circulating insulin, although measures of insulin were not conducted in the fed state and insulin sensitivity was not determined. Adiponectin is an adipose tissue‐derived signaling peptide that has been linked to insulin sensitivity in human studies, where the HMW isoform is thought to be most effective in promoting insulin sensitivity (Turer & Scherer, 2012). Similar to insulin, total adiponectin was not responsive to age or to CR; however, the HMW isoform was responsive to age and to CR. These data demonstrate that differences in survival are not associated with differences in fasting serum insulin, but may be linked to differences in adiponectin isoform distribution. In human studies, an inverse relationship between systemic levels of adiponectin and adiposity has been identified, especially in the context of obesity (Arita *et al*., 1999; Turer *et al*., 2011). In cell culture models, production of adiponectin is inversely linked to adipocyte size (Skurk *et al*., 2007). It is unclear how much this association is driven by obesity; in young nonobese mice, a relationship between circulating levels of total adiponectin and adipocyte size was not identified (Varady *et al*., 2007). Mice with deficiencies in the somatotropic axis display enhanced longevity that is associated with increased adiponectin (Bartke *et al*., 2016). The increase in adiponectin in these models is not necessarily reflected in adiposity, in particular in the GHRKO mice that have relatively high levels of visceral fat. In this study, the impact of aging on adipocyte size was similar for both control and CR tissues albeit starting from different set points: an initial shift to larger adipocytes from adult to late‐middle age and then to smaller adipocytes at advanced age. These changes were not associated with differences in total adiponectin. A relationship between HMW adiponectin and adiposity and adipocyte size was detected, but was not equivalent across the age groups. For example, even though the size distribution profiles of adipocyte size at 10 and 30 months of age were quite similar, the relationship to HMW adiponectin was completely distinct. It would be of considerable interest to investigate the relationships of adiponectin with other adipose tissues including subcutaneous and bone marrow depots where the impact of aging and CR on adiponectin production and processing may be distinct from that in the reproductive adipose depot investigated in this study.

Factors downstream of adiponectin signaling have been implicated in longevity, including AMPK, SIRT1, and PGC‐1a (Anderson & Weindruch, 2010). The inverse correlation between caloric intake and lifespan implicates nutrient‐sensitive regulators in the mechanisms of CR. The energy‐sensing kinase AMPK is a major effector in the acute response to adiponectin signaling (Okada‐Iwabu *et al*., 2013). AMPK enhances expression of NAD salvage pathway enzyme NAMPT, activating SIRT1, and positively regulates PGC‐1a (Jager *et al*., 2007; Fulco *et al*., 2008). Independent studies have shown that SIRT1 also activates PGC‐1a (Nemoto *et al*., 2005; Rodgers *et al*., 2005; Gerhart‐Hines *et al*., 2007). In adipose tissue, CR enhanced levels of all four of these metabolic regulators in a manner that suggests activation of PGC‐1a. These findings are consistent with recent work identifying mitochondrial oxidative phosphorylation and redox metabolism as part of the core response to CR in mice (Barger *et al*. 2015). In this study, we report a significant effect of age on NAD(P)H metabolism, with lower levels of NAD(P)H and changes in the chemical properties of fluorescence lifetime (τ_m_) including free (τ_1_) and bound (τ_2_) decay components. In addition, age induced an increase in a_1_, indicative of a shift away from oxidative metabolism (Bird *et al*., 2005). The impact of CR was more nuanced. The change in τ_m_ observed with CR appears not to be due to an effect on the intrinsic fluorescent properties of NAD(P)H; neither τ_1_ nor τ_2_ were altered; rather, CR induced a decrease in the ratio of free to bound NAD(P)H (a_1_), indicative of a shift toward oxidative metabolism. Differences in biochemical detection of NAD and multiphoton detection of NAD(P)H together with the age‐related changes in fluorescence decay parameters indicate a series of shifts in redox state and chemical microenvironment from adult to late‐middle age to advanced age. These data suggest that in terms of adipose tissue cellular metabolism, aging impacts NAD, redox, and the cellular microenvironment, and that the adipose tissues of CR mice are intrinsically different from those of controls.

Adipose tissues play an important role in metabolic adaptation: They are the primary energy store in the body and during fasting, provision of fatty acids as an alternate fuel source is the principal means of sparing glucose (Frayn, 2002). Apart from FFA that are adipose tissue derived, serum lipids are generally found complexed as lipoproteins that are liver derived, but peripheral tissue depleted. Thus, serum fatty acid composition is influenced by hepatic and adipose tissue lipid metabolism. Changes in the composition of circulating lipids occur as very early events in the development of spontaneous insulin resistance, and lipid signatures can accurately identify metabolic dysfunction even in the absence of differences in adiposity (Polewski *et al*., 2015). Our data show that age and CR influence the composition of circulating lipids. PL were the primary lipid class showing an impact of age. Among the 16 separate fatty acids detected, half showed a main effect of age or an age by diet interaction. It is possible that these differences influence lipoprotein function including release and uptake of lipids from lipoprotein receptors. The age‐related decrease in percent composition of 18:2n‐6 and 18:3n‐3 reported here is mirrored in studies of membrane PL composition from a range of tissues from aged rats, as is the increase in 20:4n‐6 (Merry, 2002). The impact of CR was not simply to reverse the effects of age, but extended to all lipid classes. The fatty acid composition within lipid classes appeared to have a different set point in CR mice as well as a different aging trajectory. For adipose tissue‐derived FFA, both delta 6 desaturation (16:1n‐10/16:0 and 22:6n‐3/18:3n‐3) and elongation (18:1n‐7/16:1n‐7) ratios were lower with CR. An inverse association between long‐chain FFA and longevity has been previously reported in comparative studies of mammalian longevity (Jove *et al*., 2014). These changes may be indicative of differences in FFA or TG processing in adipose tissues. Possible explanations would include reduced activities of fatty acid synthetic enzymes, or a perhaps a change in fatty acid species retention preference, or a change in selectivity of secretion processes. An emerging paradigm describes a role for lipid mediators in the association between inflammation and metabolic dysfunction (Iyer *et al*., 2010). AA is a precursor for several important proinflammatory molecules including leukotrienes, prostaglandins, and thromboxanes. Age and CR both impacted the ratio of AA to LA (20:4n‐6/18:2n‐6), suggestive of an underlying difference in inflammatory tone. Lipidomic studies in humans point to lower saturation index and lower inflammatory precursors as markers of enhanced longevity (Gonzalez‐Covarrubias *et al*., 2013). Taken together, these data point to age‐ and CR‐related differences in adipose tissue function in terms of lipid flux, and circulating factors that may contribute to systemic homeostatic mechanisms.

Our study demonstrates that aging is associated with changes in cellular metabolism in adipose tissue and in systemic metabolic parameters linked to adipose tissue function. CR had a significant impact on some but not all of these age‐related changes but also induced changes independent of age. These findings demonstrate that CR animals are metabolically distinct and are consistent with the concept that metabolism plays a role in the mechanisms of CR. Given that the benefits of CR are conserved in nonhuman primates, factors responsive to CR identified in this study may also be important in human aging and disease vulnerability (Colman *et al*., 2014). An unexpected finding from this study was the high degree of age group specificity in adipose tissue metabolism and in how adiposity relates to adipose tissue function.

## Materials and methods

### Animals

This study was approved by the Institutional Animal Care and Use Committee at the University of Wisconsin, Madison. Male B6C3F1 hybrid mice were housed under controlled pathogen‐free conditions. Mice were randomized into control or restricted groups at 2 months of age and fed 87 kcal week^−1^ (Bio‐Serv diet #F05312), which is ~95% of *ad libitum* intake, or 73 kcal week^−1^, which is a 23% reduction in calorie intake from *ad libitum* levels and 16% reduction from controls (Bio‐Serv diet #F05314). Total daily intake for CR mice was proportionately lower in carbohydrates than controls (20% lower) with the difference made up in equivalent proportional increases in fat and protein contents (Table S6, Supporting information). Fatty acid composition was identical for both diets. Mice were individually housed to ensure consumption of all food and so that precise caloric intake could be known. Body composition was determined on anesthetized mice using dual‐energy X‐ray absorptiometry (GE Lunar Piximus) 2 weeks prior tissue harvest. A list of all measured parameters for each mouse is provided in Table S7 (Supporting information).

### Multiphoton imaging

Autofluorescence detection and lifetime imaging was conducted using the multiphoton workstation at the University of Wisconsin Laboratory for Optical and Computational Instrumentation (LOCI, www.loci.wisc.edu). The system design, setup, and data acquisition have been previously described (Martin *et al*., 2015).

### Serum fatty acid composition analysis

Lipids were extracted from 100 μL of serum following a modified Folch method (Folch *et al*., 1957). Pentadecanoic acid was added as an internal control of transmethylation efficiency. Neutral lipid species were separated on silica gel‐60 TLC plates (EMD Millipore) using a heptane/isopropyl ether/acetic acid (60/40/3) solvent system. TG, CE, FFA, and PL bands were scraped from plates; lipids were extracted and transmethylated for 30 min at 100 °C with boron trifluoride in 14% methanol (Sigma). Fatty acid methyl esters were suspended in hexane and analyzed by gas liquid chromatography (GLC). Chromatograms were analyzed using HP ChemStation software. Results were calculated to express fatty acid composition as a percent of total.

### Statistical analysis

For biometric data, serum parameters including endocrine data, and lipidomics univariate measurements, two‐way ANOVA was used to estimate the effect of age and diet. Measurements were transformed to the log scale to obtain approximately normally distributed residuals. Where appropriate, *P*‐values were adjusted using the Benjamini–Hochberg method. For densitometry of Western blots, data were analyzed by Student's *t*‐test. Age effects were analyzed by one‐way ANOVA with Tukey post hoc analysis. Adipocyte medians and IQR were analyzed by two‐way ANOVA using the Holm adjustment. Adipocyte size distribution and fluorescence lifetime distribution data were binned, and the frequency in each bin was treated as a repeated measure within animal. A mixed‐effects model was fit with terms for diet, age, and bin, with a random effect for animal. Student's *t*‐test analysis of fatty acid data was adjusted using the method of Holm. Linear regression analysis was conducted to determine association between variables.

All other methods are described in the Appendix S1 (Supporting information).

## Funding

This work was supported by NIH/NIA Research Grant AG037000, NIH training fellowships DK007665 (KM), and GM083252 (PH), and the Glenn Foundation for Medical Research.

## Conflict of interest

None declared.

## Author contributions

The study was designed by JN and RA; data were generated and analyzed by KM, MB, JC, PH, MP, and TD; consultation on multiphoton imaging was provided by KE; statistical analysis was conducted ML; the paper was written by KM, MB, and RA.

## Supporting information


**Fig. S1** Serum adiponectin and fat mass.
**Fig. S2** Relationship between serum adiponectin and body weight, fat mass, and adipocyte size.
**Fig. S3** CR activates growth regulators in adipose tissue.
**Fig. S4** Aging and CR impact NAD(P)H metabolism in adipose tissue.
**Fig. S5** Ratio of essential omega‐3 and omega‐6 polyunsaturated fatty acids in diet and circulating lipids.
**Fig. S6** Impact of age and CR on serum omega‐3 to omega‐6 fatty acid index.
**Fig. S7** Impact of age and CR on elongation and desaturation indices of α‐linolenic acid (18:3n‐3) and linoleic acid (18:2n‐6).

**Table S1** Fatty acid composition of serum cholesteryl esters as percent of total.
**Table S2** Fatty acid composition of serum free fatty acids as percent of total.
**Table S3** Fatty acid composition of serum phospholipids as percent of total.
**Table S4** Fatty acid composition of serum triglycerides as percent of total.
**Table S5** Fatty acid consumption (g week^−1^).
**Table S6** Diet composition.
**Table S7** Summary of measures conducted in each animal.

**Appendix S1** Methods.
