# Supplementary figures and images for: Aging and caloric restriction impact adipose tissue, adiponectin, and circulating lipids
Source: Aging Cell. 2017 Feb 3;16(3):497–507. doi: 10.1111/acel.12575 (PMC5418198; doi:10.1111/acel.12575)

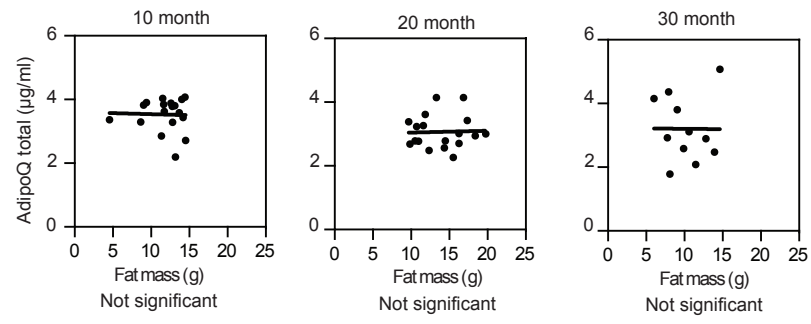

**Figure S1**

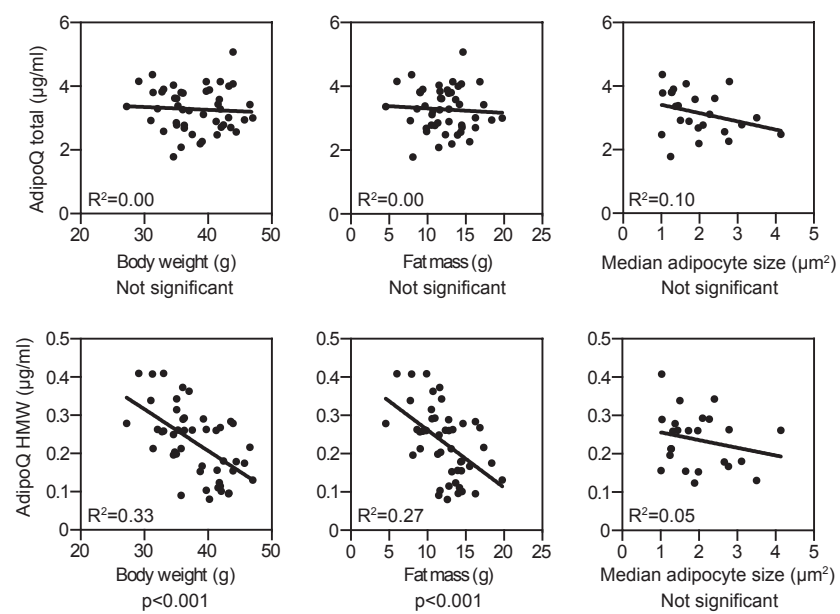

**Figure S2**

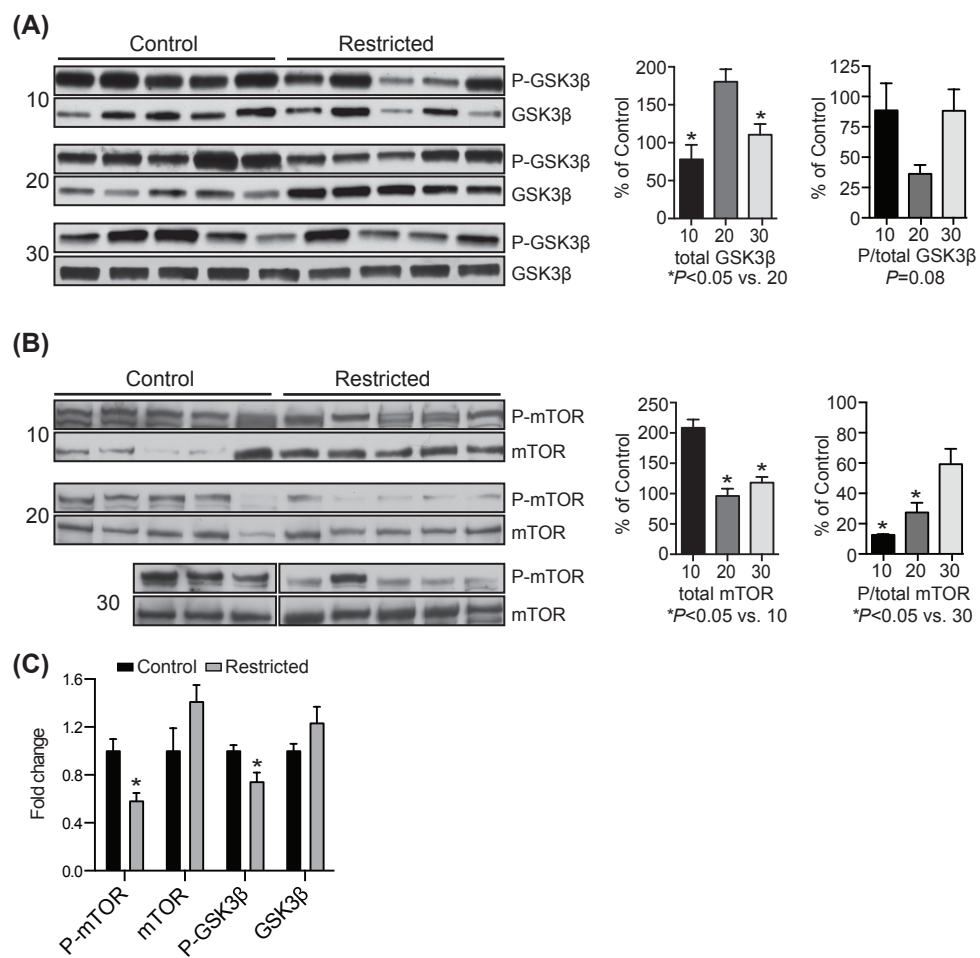

**Figure S3**

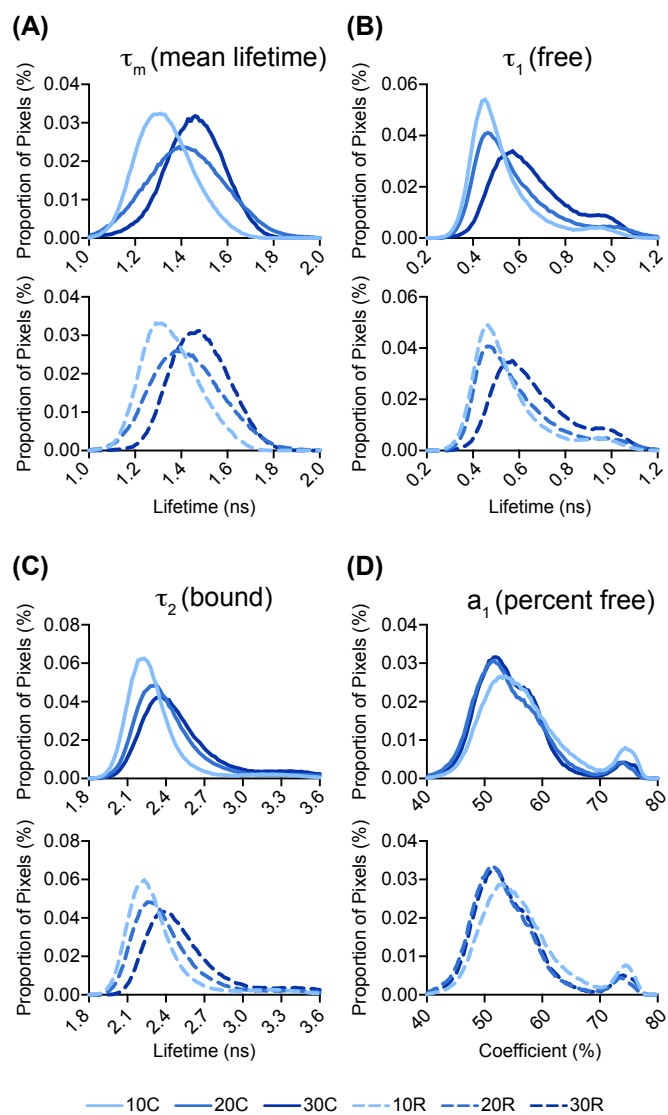

**Figure S4**

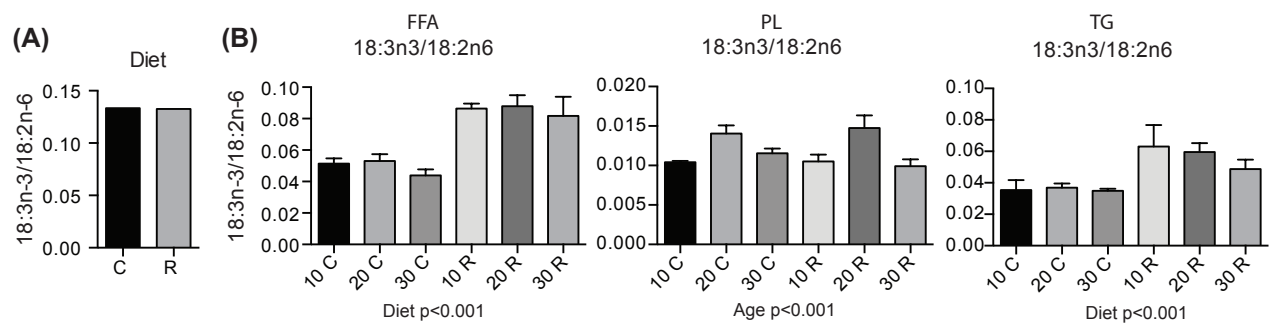

**Figure S5**

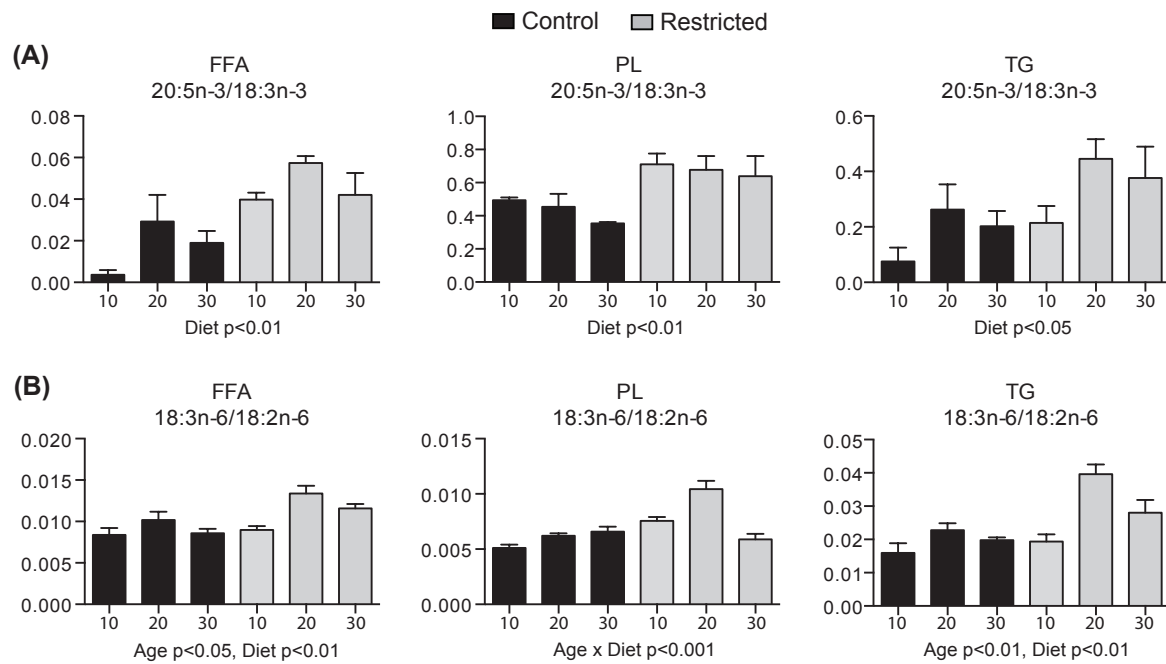

**Figure S6**

Supplement: Supplementary file 1 — Fig. S1 Serum adiponectin and fat mass. Fig. S2 Relationship between serum adiponectin and body weight, fat mass, and adipocyte size. Fig. S3 CR activates growth regulators in adipose tissue. Fig. S4 Aging and CR impact NAD(P)H metabolism in adipose tissue. Fig. S5 Ratio of essential omega‐3 and omega‐6 polyunsaturated fatty acids in diet and circulating lipids. Fig. S6 Impact of age and CR on serum omega‐3 to omega‐6 fatty acid index. Fig. S7 Impact of age and CR on elongation and desaturation indices of α‐linolenic acid (18:3n‐3) and linoleic acid (18:2n‐6). [file ACEL-16-497-s001.pdf]
