# Supplementary material for: Aging and caloric restriction impact adipose tissue, adiponectin, and circulating lipids
Source: Aging Cell. 2017 Feb 3;16(3):497–507. doi: 10.1111/acel.12575 (PMC5418198; doi:10.1111/acel.12575)
Supplement: Supplementary file 2 — Table S1 Fatty acid composition of serum cholesteryl esters as percent of total. Table S2 Fatty acid composition of serum free fatty acids as percent of total. Table S3 Fatty acid composition of serum phospholipids as percent of total. Table S4 Fatty acid composition of serum triglycerides as percent of total. Table S5 Fatty acid consumption (g week−1). Table S6 Diet composition. Table S7 Summary of measures conducted in each animal. [file ACEL-16-497-s002.pdf]

Supplemental Table 1: *Fatty acid composition of serum cholesteryl esters as percent of total*

	Control			Restricted			p-value		
	10 mo.	20 mo.	30 mo.	10 mo.	20 mo.	30 mo.	Age	Diet	Age x Diet
16:0	5.40 ± 0.65	4.53 ± 0.51	5.12 ± 0.45	4.78 ± 0.44	5.16 ± 1.48	6.67 ± 1.19			0.0485
16:1n-10	0.34 ± 0.03	0.29 ± 0.03	0.30 ± 0.00	0.40 ± 0.10	0.40 ± 0.04	0.49 ± 0.14		0.0007	
16:1n-7	2.99 ± 0.41	3.05 ± 0.34	3.22 ± 0.37	2.92 ± 0.28	3.04 ± 0.55	3.37 ± 0.59			
18:0	1.82 ± 0.37	1.31 ± 0.24	1.56 ± 0.38	1.77 ± 0.37	2.50 ± 1.37	3.37 ± 1.62			0.0211
18:1n-9	9.10 ± 1.13	8.30 ± 0.87	9.07 ± 1.24	7.76 ± 0.74	6.68 ± 0.56	9.78 ± 1.74			0.0046
18:1n-7	0.70 ± 0.10	0.58 ± 0.05	0.68 ± 0.11	0.56 ± 0.10	0.70 ± 0.34	0.86 ± 0.14			
18:2n-6	26.90 ± 2.02	23.20 ± 1.86	23.58 ± 1.13	38.76 ± 3.15	26.93 ± 2.24	32.19 ± 1.14			< 0.0001
18:3n-6	0.42 ± 0.08	0.47 ± 0.04	0.52 ± 0.04	0.68 ± 0.03	0.75 ± 0.18	0.60 ± 0.12			0.0005
20:0	0.52 ± 0.15	0.36 ± 0.12	0.34 ± 0.12	0.44 ± 0.10	0.52 ± 0.31	0.53 ± 0.14			
20:1n-9	0.05 ± 0.03	0.04 ± 0.01	0.02 ± 0.03	0.06 ± 0.02	0.09 ± 0.09	0.05 ± 0.04			
20:3n-6	0.82 ± 0.09	0.64 ± 0.11	0.66 ± 0.17	0.77 ± 0.07	0.65 ± 0.12	0.69 ± 0.08			
20:4n-6	46.20 ± 3.49	51.95 ± 2.46	50.06 ± 2.02	36.49 ± 4.06	47.53 ± 3.02	36.54 ± 4.82			< 0.0001
20:5n-3	0.50 ± 0.08	0.49 ± 0.08	0.44 ± 0.04	0.65 ± 0.05	0.71 ± 0.04	0.57 ± 0.06		< 0.0001	
22:0	0.44 ± 0.08	0.29 ± 0.10	0.26 ± 0.09	0.36 ± 0.08	0.52 ± 0.41	0.51 ± 0.05			
22:6n-3	3.81 ± 0.61	4.51 ± 0.29	4.17 ± 0.51	3.60 ± 0.44	3.91 ± 0.32	3.79 ± 0.16			

Data are mean ± SD

Supplemental Table 2: *Fatty acid composition of serum free fatty acids as percent of total*

	Control			Restricted			p-value		
	10 mo.	20 mo.	30 mo.	10 mo.	20 mo.	30 mo.	Age	Diet	Age x Diet
16:0	30.50 ± 0.48	29.32 ± 1.48	31.48 ± 2.28	29.11 ± 0.83	28.84 ± 1.18	29.59 ± 2.27			
16:1n-10	0.65 ± 0.07	0.67 ± 0.10	0.57 ± 0.10	0.46 ± 0.03	0.49 ± 0.04	0.50 ± 0.05		< 0.0001	
16:1n-7	8.24 ± 0.50	8.42 ± 1.62	7.51 ± 0.96	10.20 ± 0.97	10.01 ± 1.45	8.25 ± 1.75			
18:0	11.45 ± 1.26	10.45 ± 1.46	10.59 ± 1.53	9.34 ± 1.43	9.05 ± 0.77	9.52 ± 2.11			
18:1n-9	27.61 ± 0.67	29.12 ± 3.25	26.72 ± 3.34	26.43 ± 1.27	25.37 ± 1.94	26.68 ± 2.78			
18:1n-7	2.90 ± 0.21	2.71 ± 0.32	2.60 ± 0.20	2.38 ± 0.19	2.27 ± 0.21	2.63 ± 0.18			0.0088
18:2n-6	11.86 ± 0.90	11.99 ± 1.09	12.66 ± 0.91	15.50 ± 1.59	16.32 ± 1.52	16.14 ± 1.25		< 0.0001	
18:3n-6	0.10 ± 0.02	0.12 ± 0.02	0.11 ± 0.02	0.14 ± 0.02	0.22 ± 0.05	0.19 ± 0.03	0.0068	< 0.0001	
18:3n-3	0.61 ± 0.09	0.63 ± 0.09	0.56 ± 0.11	1.34 ± 0.19	1.42 ± 0.18	1.33 ± 0.48		< 0.0001	
20:0	0.48 ± 0.12	0.39 ± 0.13	0.28 ± 0.04	0.36 ± 0.06	0.36 ± 0.15	0.36 ± 0.08			
20:1n-9	0.38 ± 0.06	0.39 ± 0.08	0.28 ± 0.04	0.40 ± 0.05	0.28 ± 0.04	0.38 ± 0.13			
20:3n-6	0.45 ± 0.10	0.38 ± 0.20	0.43 ± 0.16	0.38 ± 0.09	0.29 ± 0.07	0.33 ± 0.07			
20:4n-6	3.79 ± 0.54	4.20 ± 1.64	4.81 ± 1.26	2.85 ± 0.53	3.70 ± 0.86	2.82 ± 0.71			
20:5n-3	ND	0.02 ± 0.02	0.01 ± 0.01	0.05 ± 0.01	0.08 ± 0.02	0.05 ± 0.02		< 0.0001	
22:0	0.24 ± 0.07	0.20 ± 0.07	0.16 ± 0.06	0.18 ± 0.02	0.22 ± 0.11	0.23 ± 0.05			
22:6n-3	0.73 ± 0.08	0.98 ± 0.39	1.23 ± 0.41	0.88 ± 0.12	1.06 ± 0.22	1.01 ± 0.16			

ND, not detected; Data are mean ± SD

Supplemental Table 3: *Fatty acid composition of serum phospholipids as percent of total*

	Control			Restricted			p-value		
	10 mo.	20 mo.	30 mo.	10 mo.	20 mo.	30 mo.	Age	Diet	Age x Diet
16:0	27.09 ± 1.01	27.19 ± 2.76	28.37 ± 1.72	27.31 ± 1.66	26.86 ± 1.24	28.19 ± 0.82			
16:1n-10	0.32 ± 0.14	0.29 ± 0.06	0.30 ± 0.11	0.28 ± 0.07	0.25 ± 0.06	0.24 ± 0.10			
16:1n-7	0.74 ± 0.19	0.82 ± 0.05	0.89 ± 0.05	0.78 ± 0.04	0.80 ± 0.11	0.81 ± 0.19			
18:0	18.96 ± 1.29	18.45 ± 1.31	18.73 ± 0.90	17.51 ± 0.52	18.30 ± 0.69	17.44 ± 1.26			
18:1n-9	11.54 ± 1.45	10.74 ± 0.89	10.78 ± 0.39	10.90 ± 0.40	8.62 ± 0.95	9.88 ± 0.74	0.0099	0.0042	
18:1n-7	2.96 ± 0.40	2.80 ± 0.70	2.68 ± 0.41	2.56 ± 0.23	2.45 ± 0.62	3.16 ± 0.43			
18:2n-6	14.41 ± 0.86	13.41 ± 0.50	13.78 ± 0.81	18.84 ± 1.60	16.12 ± 0.83	18.02 ± 1.61	0.0145	< 0.0001	
18:3n-6	0.07 ± 0.01	0.08 ± 0.01	0.09 ± 0.02	0.14 ± 0.01	0.17 ± 0.03	0.11 ± 0.02			< 0.0001
18:3n-3	0.15 ± 0.01	0.19 ± 0.03	0.16 ± 0.02	0.20 ± 0.03	0.24 ± 0.06	0.18 ± 0.04	0.0665	0.0211	
20:0	0.18 ± 0.02	0.18 ± 0.02	0.17 ± 0.02	0.18 ± 0.01	0.19 ± 0.03	0.16 ± 0.03			
20:1n-9	0.36 ± 0.05	0.37 ± 0.09	0.35 ± 0.05	0.31 ± 0.05	0.33 ± 0.07	0.41 ± 0.04			
20:3n-6	2.34 ± 0.07	1.76 ± 0.39	1.49 ± 0.19	2.15 ± 0.15	1.45 ± 0.40	1.65 ± 0.07	0.0004		
20:4n-6	15.09 ± 1.90	17.09 ± 0.68	16.22 ± 0.67	12.89 ± 1.64	17.20 ± 0.56	13.38 ± 2.14	0.0026	0.0224	
20:5n-3	0.07 ± 0.01	0.08 ± 0.02	0.06 ± 0.01	0.14 ± 0.03	0.15 ± 0.01	0.11 ± 0.03	0.0030	< 0.0001	
22:0	0.20 ± 0.03	0.21 ± 0.05	0.18 ± 0.04	0.21 ± 0.03	0.18 ± 0.03	0.16 ± 0.02			
22:6n-3	5.51 ± 0.63	6.34 ± 0.25	5.77 ± 0.34	5.59 ± 0.69	6.69 ± 0.34	6.10 ± 0.28	0.0028		

Data are mean ± SD

Supplemental Table 4: *Fatty acid composition of serum triglycerides as percent of total*

	Control			Restricted			p-value		
	10 mo.	20 mo.	30 mo.	10 mo.	20 mo.	30 mo.	Age	Diet	Age x Diet
16:0	31.14 ± 3.38	27.92 ± 2.18	28.12 ± 1.70	26.30 ± 1.11	26.74 ± 2.41	26.45 ± 2.45			
16:1n-10	0.55 ± 0.23	0.74 ± 0.09	0.58 ± 0.12	0.46 ± 0.08	0.48 ± 0.06	0.45 ± 0.10			
16:1n-7	2.43 ± 0.96	3.17 ± 0.53	3.05 ± 0.34	2.49 ± 0.40	2.93 ± 0.61	2.95 ± 0.56			
18:0	14.15 ± 4.97	10.09 ± 2.42	10.10 ± 1.19	12.11 ± 1.72	10.33 ± 3.23	10.33 ± 2.32			
18:1n-9	28.77 ± 7.39	33.19 ± 3.62	33.18 ± 2.91	25.80 ± 5.13	23.79 ± 4.05	28.96 ± 1.57			
18:1n-7	2.39 ± 0.71	2.24 ± 0.37	2.12 ± 0.24	2.12 ± 0.46	1.57 ± 0.10	2.34 ± 0.26			
18:2n-6	9.86 ± 3.06	12.86 ± 2.61	13.71 ± 1.55	15.72 ± 1.85	18.11 ± 3.75	16.71 ± 3.84		0.0012	
18:3n-6	0.16 ± 0.10	0.30 ± 0.09	0.27 ± 0.05	0.31 ± 0.07	0.72 ± 0.22	0.46 ± 0.13	0.0010	< 0.0001	
18:3n-3	0.35 ± 0.20	0.48 ± 0.16	0.48 ± 0.10	0.97 ± 0.38	1.07 ± 0.30	0.82 ± 0.26		< 0.0001	
20:0	4.70 ± 2.54	3.12 ± 1.50	2.82 ± 0.65	3.36 ± 0.89	2.96 ± 1.77	2.63 ± 0.44			
20:1n-9	0.62 ± 0.20	0.61 ± 0.18	0.52 ± 0.08	0.83 ± 0.19	0.41 ± 0.21	1.00 ± 0.53			
20:3n-6	0.30 ± 0.03	0.29 ± 0.06	0.29 ± 0.02	0.48 ± 0.04	0.31 ± 0.04	0.48 ± 0.17			0.0005
20:4n-6	1.11 ± 0.40	1.80 ± 0.40	1.83 ± 0.43	5.40 ± 3.40	6.62 ± 2.57	3.09 ± 1.46			0.0001
20:5n-3	0.03 ± 0.04	0.12 ± 0.09	0.10 ± 0.07	0.18 ± 0.07	0.45 ± 0.12	0.28 ± 0.21	0.0514	0.0011	
22:0	3.17 ± 1.83	2.15 ± 1.05	1.97 ± 0.51	2.54 ± 0.78	2.27 ± 1.42	1.89 ± 0.25			
22:6n-3	0.28 ± 0.17	0.90 ± 0.40	0.83 ± 0.25	0.95 ± 0.33	1.24 ± 0.34	1.15 ± 0.72			0.0007

Data are mean ± SD

Supplemental Table 5. *Fatty acid consumption (g/week)*

Fatty acid	Control (g/week)	percent	Restricted (g/week)	percent
16:0 Palmitic	0.099	11%	0.138	11%
18:0 Stearic	0.042	5%	0.058	5%
18:1 Oleic	0.213	24%	0.297	24%
18:2n-6 Linoleic	0.480	53%	0.671	54%
18:3n-3 Linolenic	0.064	7%	0.089	7%
20:0 Eicosanoic	0.003	0%	0.000	0%
22:0 Behenic	0.003	0%	0.000	0%
Total:	0.904		1.254	

Supplemental Table 6: *Diet composition*

	Control (% Kcal)	Restricted (% Kcal)
Protein	13.8	22.6
Fat	9.7	16.6
Carbohydrate	76.5	60.8

Supplemental Table 7: *Summary of measures conducted in each animal*

Group	Animal	Insulin	Adiponectin	Morpho.	Western blot	NAD	FLIM	Serum FA
10 mo. Control	C-1	x	x	x		x		
	C-2			x				x
	C-3	x	x	x		x	x	
	C-4	x	x	x	x	x	x	x
	C-5	x	x		x		x	
	C-6	x	x			x	x	
	C-7		x		x			x
	C-8		x		x	x		x
	C-9	x	x					
	C-10		x		x	x	x	x
10 mo. Restricted	CR-1	x	x		x			x
	CR-2	x	x		x	x		
	CR-3	x	x		x			
	CR-4					x		x
	CR-5	x	x	x			x	
	CR-6	x	x	x	x	x	x	
	CR-7		x	x	x	x	x	x
	CR-8	x	x			x		
	CR-9		x	x			x	x
	CR-10		x			x		x
20 mo. Control	C-1	x	x					x
	C-2		x	x	x	x	x	x
	C-3		x		x	x		
	C-4	x	x		x		x	x
	C-5	x	x	x			x	x
	C-6	x	x			x		x
	C-7		x		x			
	C-8		x	x	x	x	x	
	C-9		x	x		x	x	
	C-10							
20 mo. Restricted	CR-1		x	x		x	x	
	CR-2	x	x		x	x	x	x
	CR-3	x	x	x	x		x	x
	CR-4			x	x	x	x	x
	CR-5	x	x	x		x		
	CR-6	x	x	x		x		x
	CR-7	x	x		x			
	CR-8		x	x				
	CR-9		x	x	x	x		x
	CR-10		x				x	
30 mo. Control	C-12		x	x		x	x	x
	C-15			x	x	x	x	x
	C-17	x	x	x	x	x	x	x
	C-19	x	x	x	x	x	x	x
	C-20	x	x		x	x	x	x
30 mo. Restricted	CR-11		x				x	
	CR-12				x			x
	CR-13	x	x	x	x	x	x	x
	CR-15	x	x	x	x		x	
	CR-16	x	x		x	x	x	
	CR-17	x	x		x	x		x
	CR-19	x	x	x				x
	CR-20	x	x	x		x	x	x

Body weight and body composition were measured in all mice.
