# Supplementary material for: Aging and caloric restriction impact adipose tissue, adiponectin, and circulating lipids
Source: Aging Cell. 2017 Feb 3;16(3):497–507. doi: 10.1111/acel.12575 (PMC5418198; doi:10.1111/acel.12575)
Supplement: Supplementary file 3 — Appendix S1 Methods. [file ACEL-16-497-s003.docx]

*Supplemental Information*

**Supplemental Methods**

*Animals:* This study was approved by the Institutional Animal Care and Use Committee at the University of Wisconsin, Madison. Male B6C3F1 hybrid mice were housed under controlled pathogen-free conditions. Mice were randomized into control or restricted groups at 2 months of age and fed 87 kcal/wk (Bio-Serv diet #F05312) which is ~95% of ad libitum intake, or 73 kcal/wk, which is a 23% reduction in calorie intake from ad libitum levels and 16% reduction from controls (Bio-Serv diet #F05314). The CR diet was originally designed to be isocaloric with the Control diet under a more stringent restriction intervention, the resulting total daily intake for CR mice was proportionately lower in carbohydrates than Controls (20% lower) with the difference made up in equivalent proportional increases in fat and protein. Lipid composition was identical for both diets (Table S1). Mice were individually housed to ensure consumption of all food and that precise caloric intake could be known. Body composition was determined on anesthetized mice using dual energy x-ray absorptiometry (GE Lunar Piximus) two weeks prior tissue harvest. Mice were euthanized by cervical dislocation at 10, 20 or 30 months of age. Tissues were isolated, fixed in formalin and paraffin embedded or flash frozen in liquid nitrogen and stored at -80 °C until further processing.

*Histology, morphometrics:* Hematoxylin and eosin staining was conducted on 10um sections from paraffin-embedded epididymal adipose tissues using standard techniques. Images were captured using Leica DM4000B microscope with Retiga 4000R digital camera (QImaging Systems, Surrey, BC, Canada). Image analysis was performed for n=4-7 animals per group and 5 images per animal using ImagePro Plus 6.3.1.542 Software. For each field adipocyte size was detected and categorized into bins, and distributions compiled. Data were analyzed by three way ANOVA including age, diet, and adipocyte size (6 bins).

*Protein detection ELISA and western blot:* Commercially available ELISA kits were used to detect circulating levels of insulin (80-INSMS-E01; Alpco) and adiponectin (DY1119; R&D Systems). High molecular weight adiponectin isoforms were distinguished from total adiponectin levels by proteinase K digest. Immunodetection by western blot was performed using standard techniques as previously described ([Anderson *et al.* 2008](#_ENREF_1)) using the following antibodies: PGC-1a (sc-13067; Santa Cruz Biotechnology), SIRT1 (A13973; Life Technologies), NAMPT (V9139; Sigma Aldrich), AMPKa (2532; Cell Signaling Technologies); pAMPKa (thr172) (2535; Cell Signaling Technologies), pGSK-3b (ser9) (9336; Cell Signaling Technologies), GSK-3b (9315; Cell Signaling Technologies), TBP (8515; Cell Signaling Technologies), pmTOR (ser2448) (2971; Cell Signaling Technologies), mTOR (2972; Cell Signaling Technologies). Densitometry was performed using Adobe Photoshop CC (Adobe Systems, San Jose, CA).

*Fluorescence Lifetime:* Briefly, paraffin sections of epididymal adipose tissue (10 um) were placed on glass slides, deparafinized, and mounted with glass coverslip with Permount mounting solution. The instrument response function of the optical system was calibrated before each imaging session (Bird et al., 2004). A Nikon CFI Plan Apo 20X lens (Melville, NY, USA) was used for all imaging. Data were collected using an excitation wavelength of 780 nm, and emission was filtered at 457±50 nm, the spectral peak for NADH/NADPH. The data collection time was 120 s using a pixel frame size of 256 X 256. The system has multiple detectors including a 16 channel combined spectral lifetime detector (Hamamatsu PML-16 PMT), detection range 350 to 720 nm, and a H7422P GaAsP photon counting PMT (Hamamatsu) for intensity and lifetime imaging. Acquisition was performed with WiscScan, a LOCI developed acquisition package software. Autofluorescence intensity and fluorescence lifetime data were analyzed in SPCImage (Becker & Hickl, v.2.9.1, Berlin, Germany) where a Levenberg–Marquardt routine for nonlinear fitting was used to fit the fluorescence decay curve collected for each pixel in the 256 X 256 frame to a model multi-exponential decay function using a two component fit. Mean lifetime is calculated as: τ_m_ = a_1_• τ_1_ + a_2_• τ_2._ Data were assessed by the minimized chi-square value generated during the fit so that analysis was unbiased. To eliminate background fluorescence a threshold for analysis was applied based on photon counts. Data were binned to maintain high quality of the fit. For autofluorescence intensity, data were analyzed in ImageJ (NIH, Wayne Rasband, http://rsb.info.nih.gov/ij/).

*Biochemical assay for NAD:* Levels of total NAD were detected using a commercially available kit (#K337; BIovision). Extracts from ~300 ug of adipose tissue were purified using 10 kDa size exclusion columns (#1977; Biovision) to remove proteins, and levels of NAD quantified according to the manufacturer’s instructions.

**Supplemental Figure Legends**

Figure S1: Serum adiponectin and fat mass. Linear regression analyses of serum adiponectin and fat mass in 10 (n=18), 20 (n=18), and 30 (n=11) month old mice, includes Control and Restricted animals at each age.

Figure S2: Relationship between serum adiponectin and body weight, fat mass, and adipocyte size. Linear regression analyses of serum total (top row) or HMW (bottom row) adiponectin were performed with body weight (left, n=47), fat mass (middle, n=47), or median adipocyte size (right, n=24).

Figure S3: CR activates growth regulators in adipose tissue. (A) Detection of levels of GSk3b and phospho-GSK3b (serine 9) n=4-5 per group. (B) Detection of levels of mTOR and phospho-mTOR (serine 2448) n=3-5 per group. (C) Quantitation of protein levels across all age groups, n=13-15 per age group. Data are shown as means ± SEM, asterisk(*) indicates p<0.05. Significance determined by Student’s t-test.

Figure S4: Aging and CR impact NAD(P)H metabolism in adipose tissue. (A) fluorescence lifetime distributions (λ_ex_=780nm) of τ_m_, (B) τ_1_, (C) τ_2_, (D) a_1_, for adipose tissues from control and CR mice at indicated ages n=4-5 per age per diet.

Figure S5: Ratio of essential omega-3 and omega-6 polyunsaturated fatty acids in diet and circulating lipids. The ratio of the essential fatty acids α-linolenic acid (18:3n-3) and linoleic acid (18:2n-6) in (A) Control and CR diets (provided by manufacturer), (B) circulating lipids including free fatty acids (FFA), phospholipids (PL), and triglycerides (TG). Data shown are mean ± SEM, n=5 per group.

Figure S6: Impact of age and CR on serum omega-3 to omega-6 fatty acid index.

Serum ratio of 20:5n-3 to 20:4n-6 in cholesterol esters (CE), free fatty acids (FFA), phospholipids (PL), and triglycerides (TG). Data shown are median with IQR, n=5 per group. Significance determined by 2-way ANOVA.

Figure S7: Impact of age and CR on elongation and desaturation indices of α-linolenic acid (18:3n-3) and linoleic acid (18:2n-6). Serum ratio of (A) omega-3 fatty acids 20:5n-3 to 18:3n-3 and (B) omega-6 fatty acids 18:3n-6 to 18:2n-6 in free fatty acids (FFA), phospholipids (PL), and triglycerides (TG). Data shown are mean ± SEM, n=5 per group. Significance determined by 2-way ANOVA.
